# Relationship between lipid profiles and plasma total homocysteine, cysteine and the risk of coronary artery disease in coronary angiographic subjects

**DOI:** 10.1186/1476-511X-10-137

**Published:** 2011-08-12

**Authors:** Yunjun Xiao, Yuan Zhang, Xiaofei Lv, Dongfang Su, Dan Li, Min Xia, Jian Qiu, Wenhua Ling, Jing Ma

**Affiliations:** 1 Guangdong Provincial Key Laboratory of Food, Nutrition and Health; Department of Nutrition, School of Public Health, Sun Yat-sen University. 510080, Number 74 Zhongshan Road 2, Guangzhou, Guangdong, PR of China; 2 Department of Cardiology, Guangzhou Military General Hospital. 510010, Number 111 Liuhua Road, Guangzhou, Guangdong, PR of China

**Keywords:** Homocysteine, Cysteine, Lipid profiles, Coronary artery disease

## Abstract

**Background:**

Homocysteine and cysteine are considered as risk factors of cardiovascular disease. Homocysteine influences the liver expression of ApoA-I and decreases its blood level and HDL in genetic mice model. We aimed therefore to evaluate whether homocysteine and cysteine are associated with lipid parameters, and the joint effects of them on the risk of coronary artery disease (CAD). Plasma total homocysteine (tHcy), cysteine (tCys) and lipid markers were measured in 2058 consecutive coronary artery angiographic patients.

**Results:**

Plasma tHcy but not tCys correlated negatively with ApoA-I (r = -0.153, *P *< 0.001) and with HDL cholesterol (r = -0.148, *P *< 0.001), and correlated positively with the risk of CAD (OR: 1.61; 95% confidence interval; 1.26 to 2.05). Combination of high tHcy and high tCys levels was associated with decreased ApoA-I and HDL cholesterol levels, and with increased risk of CAD (OR: 1.696, 95% CI (1.301-2.211)). Furthermore, low HDL cholesterol combined with low tHcy or high tHcy all had increased risk for CAD (OR: 1.254, 95% CI (1.114-1.565); OR: 1.332, 95% CI (1.093-1.624); respectively) whereas high HDL cholesterol counteracted the harmful effect of high tHcy on the risk of CAD. However, only the combination of high tHcy and high ApoA-I had an increased risk for CAD (OR: 1.438, 95% CI (1.170-1.768)).

**Conclusions:**

The association of homocysteine and cysteine, ApoA-I or HDL cholesterol and their joint effects provide new insights on its role on CAD.

## Introduction

Hyperhomocysteinemia has been considered as an independent risk factor of coronary artery disease (CAD)[1,2], but recent several large scale intervention studies found lowering the plasma total homocysteine (tHcy) with folic acid, vitamin B_6 _and B_12 _did not reduce the risk of cardiovascular disease[3]. Thus the cause-effect relationship of homocysteine and cardiovascular disease is controversial [4,5]. Furthermore, another sulf-containing amino acid cysteine, structurally like to homocysteine, was reported to be a risk factor of cardiovascular disease[6-8], but in prospective study, plasma total cysteine (tCys) was not an independent risk factor of cardiovascular disease[9]. The atherogenicity of homocysteine may involve several mechanisms including LDL-cholesterol oxidative modification, and HDL-cholesterol decrease [10]. Several studies reported homocysteine inhibited ApoA-I protein expression and decreased HDL cholesterol levels in vitro and animal model [11,12]. Cysteine is a vital structural and functional component of ApoB, the protein of LDL [13,14]. Though less reactive than homocysteine, cysteine exhibits autooxidation properties in the presence of metal ions which can support superoxide-mediated modification of LDL, thus facilitating foam cell formation [15]. But the relationship between plasma tHcy, tCys levels and lipid profiles in CAD patients are still uncertain. Here, we investigated the relationship between plasma tHcy, tCys and the lipid parameters, and the joint effects of them on the risk of CAD.

## Methods

### Subjects

The present cross-sectional study includes a total of 2,058 consecutive patients 40 to 85 years of age who had undergone a diagnostic coronary angiography at 3 hospitals (Guangzhou Military General Hospital, Sun Yat-Sen Memorial Hospital and Zhujiang Hospital) during December 2008 to September 2010 in Guangzhou, China. Those with medical illnesses such as acute infection, chronic hepatic dysfunction or nutritional derangements, malignancies, and other severe medical illnesses were excluded. All patients were free of drugs which would influence the plasma homocysteine levels, including folate or multivitamins. Of the 2,058 patients, The CAD patients (n = 1053) were defined as having significant stenosis in ≥ 1 major coronary artery and those (n = 1005) who did not have significant stenosis of all arteries were defined as controls. Any instances of concomitant illness and any current medications were documented among our study subjects. We recorded 209 cases (10%) with stroke, 108 individuals (5.2%) with atrial fibrillation, 221 cases (10.6%) of arrhythmia. The patients were accepted different medications that were 59.7% subjects who used statins; 60.3% patients used aspirin; 43% patients used beta-receptor blocker; 25% patients used angiotensin-converting enzyme inhibitor; 20% subjects used nitrates. All patients were gave informed consent to provide blood samples and the study was approved by hospitals ethics committee.

### Coronary angiography

Coronary angiographies were performed using a standard Judkins technique through the femoral artery or brachial artery. The angiograms were interpreted by two or more independent cardiologists in a blind fashion. All evaluations were based on the American Heart Association method[16]. CAD was defined as diameter stenosis ≥ 50% in the left main, left anterior descending, left circumflex, and/or right coronary artery.

### Biochemical measurement

After the patients had fasted overnight, blood samples were drawn into EDTA-containing tubes by venipuncture. Samples were immediately placed on ice and transported to the laboratory. Plasma and serum were prepared and stored at -80°C until analysis. The plasma tHcy and tCys, which include the sum of protein-bound and free homocysteine and cysteine, were simultaneously measured by high-performance liquid chromatography with fluorescence detection[17]. The total serum cholesterol, triglyceride, and HDL cholesterol concentrations were determined enzymatically. LDL cholesterol was assayed using an indirect method. Apo A-I and ApoB were simultaneously measured by immunoassay.

### Statistical analysis

Data are presented as medians and interquartile ranges for skewed variables. Unless otherwise indicated, values are expressed as mean±SD or as percentages for categorical variables. Comparisons between groups were performed using Kruskal-Wallis test followed where relevant by Mann-Whitney *U *test with adjustment for multiple comparisons (continuous variables) or the chi-square test (categorical variables). Correlations between selected pairs of variables were evaluated with the spearman correlation and partial correlation with adjustment for age, gender and other factors. The tHcy and tCys were divided into quartiles for analysis. General linear model analysis was performed to evaluate the relationship between tHcy, tCys and the lipid profiles. In multiple logistic regressions, CAD was considered as a dependent variable, with appropriate adjustment for covariates.

The analyses were also performed for different combinations of low (≤12 μmol/L), high (>12 μmol/L) tHcy or low (<219.5 μmol/L), medium (219.5-284.1 μmol/L), high (>284.1 μmol/L) tCys. Subjects with combinations of low levels of tHcy and tCys served as the reference group. To evaluate joint effect of tHcy and HDL or ApoA-I on the risk of CAD, we also performed multiple logistic regressions and adjustment for other covariates with different combinations of low, high tHcy levels and high (>1.05 mmol/L), low (≤1.05 mmol/L) HDL cholesterol or high (>1.17 mg/L), low (≤1.17 mg/L) ApoA-I. Subjects with combinations of low levels of tHcy and high levels of HDL cholesterol or ApoA-I served as the reference group. Two-side *P *values below 0.05 were considered to indicate statistical significance. All statistical analyses were performed using SPSS 13.0 software (SPSS Inc., Chicago, Illinois).

## Results

### Clinical characteristics of subjects

Table 1 shows the study population characteristics stratified by gender and presence or absence of CAD, 61% of the study population was male, and 51% had CAD. Mean age was 62.4 ± 12.5 years in the four subgroups. Plasma triglycerides, HDL cholesterol, ApoA-I, fasting plasma glucose were different in CAD cases relative to control, as were plasma concentrations of tHcy and creatinine. Plasma tCys levels were only increased in CAD cases compared to control in males.

**Table 1:** Demographic and clinical characteristics of the study population *^, †^

Characteristics	Men		Women	
		
	Control (n = 562)	CAD (n = 693)	Control (n = 443)	CAD (n = 360)
Age, yrs ^‡, §^	58.1 ± 14.6	63.7 ± 11.4 ^||^	61.4 ± 12.5	68.1 ± 10.2 ^||^
BMI, kg/m^2^	24.3 ± 4.32	24.7 ± 4.01	24.2 ± 4.33	24.6 ± 3.41
Smokers ^‡, §^	186(33.1%)	253(36.5%) ^||^	7(1.6%)	10(2.8%)
Hypertension ^‡, §^	315(56.0%)	421(60.8%)	269(60.7%)	255(70.8%) ^||^
Positive family history	44(7.8%)	31(4.5%)	30(6.8%)	30(8.3%)
Total cholesterol, mmol/L ^‡, §^	4.63 ± 1.03	4.57 ± 1.03	4.91 ± 1.06	5.08 ± 1.09 ^||^
Triglycerides, mmol/L	1.82 ± 1.24	1.93 ± 1.27 ^||^	1.74 ± 1.13	1.95 ± 1.19 ^||^
LDL cholesterol, mmol/L ^§^	2.95 ± 0.90	2.92 ± 0.94	3.04 ± 0.94	3.16 ± 0.97
HDL cholesterol, mmol/L ^‡, §^	1.06 ± 0.29	1.03 ± 0.34 ^||^	1.22 ± 0.30	1.18 ± 0.29 ^||^
ApoA-I, mg/L ^‡^	1.18 ± 0.44	1.11 ± 0.32 ^|| ^	1.31 ± 0.46	1.25 ± 0.26 ^||^
ApoB, mg/L	0.77(0.64-0.88)	0.76(0.61-0.89)	0.78(0.64-0.90)	0.80(0.65-0.97)
LpA, mg/L	0.32(0.25-0.44)	0.34(0.24-0.47)	0.33(0.26-0.41)	0.34(0.26-0.45)
LDL cholesterol/ApoB ratio	3.87(3.46-4.23)	3.81(3.42-4.21)	3.88(3.45-4.29)	3.82(3.51-4.21)
Fasting plasma glucose, mmol/L	5.80 ± 2.23	6.19 ± 2.43 ^||^	5.98 ± 2.45	6.32 ± 2.81 ^||^
Creatinine, μmol/L ^‡^	87.5(73.0-104)	89.0(76.0-104) ^||^	69.0(56.0-85.0)	71.0(58.0-89.0) ^||^
tHcy, μmol/L ^‡, §^	13.8 ± 5.93	14.5 ± 6.13 ^||^	12.3 ± 5.53	12.9 ± 6.08 ^||^
tCys, μmol/L	248.4 ± 46.3	255.8 ± 48.3 ^||^	254.2 ± 47.6	251.3 ± 45.5

* Values are mean ± SD, n (%), or median (interquartile range)

† BMI and plasma variables in the four groups were compared by Kruskal-Wallis test followed where relevant by Mann-Whitney *U *test with adjustment for multiple comparisons. *P *< 0.0125(0.05/4) was considered significant.

‡ Significantly different in men compared to women in the no CAD group.

§ Significantly different in men compared to women in the CAD group.

|| Significantly different compared to no CAD group within the same gender.

### Relationship between lipid profiles and plasma tHcy, tCys

In spearman analysis (Table 2), plasma tHcy correlated negatively with plasma HDL cholesterol and Apo A-I levels (r = -0.148, *P *< 0.001 and r = -0.153, *P *< 0.001, respectively). By using covariance analyses (ANCOVA), the plasma HDL cholesterol and ApoA-I levels were found stepwise decreasing from lowest quartile to highest quartile of tHcy after adjusted for age, gender and other confounders (all *P *< 0.001 for trend) (Table 3).

**Table 2:** Correlation coefficients of plasma tHcy, tCys and other characteristics

	tHcy (μmol/L)				tCys (μmol/L)			
		
	r*	*P*	*r*^† ^	*P*	r*	*P*	*r*^† ^	*P*
BMI, kg/m^2 ^	-0.021	0.684	0.038	0.467	-0.014	0.790	0.022	0.677
Total cholesterol, mmol/L	-0.020	0.354	0.023	0.666	-0.008	0.707	0.033	0.533
Triglycerides, mmol/L	-0.035	0.112	0.058	0.269	-0.004	0.866	0.045	0.386
LDL cholesterol, mmol/L	-0.001	0.963	0.011	0.841	-0.010	0.648	0.018	0.728
HDL cholesterol, mmol/L	-0.148	**<0.001**	-0.137	**0.009**	-0.003	0.891	0.022	0.669
ApoA-I, mg/L	-0.153	**<0.001**	-0.135	**0.010**	0.029	0.189	0.018	0.735
ApoB, mg/L	0.002	0.946	0.002	0.965	-0.025	0.253	0.055	0.296
LpA, mg/L	0.021	0.331	-0.019	0.715	0.004	0.850	-0.053	0.316
LDL cholesterol/ApoB ratio	-0.009	0.689	0.249	**<0.001**	0.026	0.232	0.042	0.427
Fasting plasma glucose, mmol/L	-0.009	0.677	0.040	0.441	-0.016	0.476	-0.067	0.199
Creatinine, μmol/L	0.207	**<0.001**	0.228	**<0.001**	-0.011	0.629	0.026	0.616

* Correlation between selected paired variables was analysis with spearman correlation.

† Correlation between selected paired variables was analysis with partial correlation adjusted for age, gender, smoke, hypertension, positive family history and coronary artery disease status.

Statistics for variables significantly associated with tHcy or tCys at *P *< 0.05 are shown in bold.

**Table 3:** Lipid profiles according to the quartiles of plasma tHcy and tCys levels *

Variable	Q1	Q2	Q3	Q4	*P *for trend
tHcy (μmol/L)	<9.1	9.1-12.3	12.4-16.5	>16.5	
Total cholesterol, mmol/L	4.66(4.57-4.76)	4.81(4.72-4.89)	4.73(4.64-4.82)	4.79(4.69-4.88)	0.126
Triglycerides, mmol/L	1.89(1.77-2.02)	1.78(1.66-1.90)	1.93(1.81-2.05)	1.85(1.73-1.97)	0.383
LDL cholesterol, mmol/L	2.93(2.85-3.01)	3.03(2.95-3.11)	3.00(2.92-3.08)	3.04(2.96-3.12)	0.266
HDL cholesterol, mmol/L	1.15(1.14-1.17)	1.14(1.13-1.16)	1.08(1.07-1.10)	1.05(1.03-1.06)	**<0.001**
ApoA-I, mg/L	1.26(1.24-1.28)	1.23(1.21-1.25)	1.19(1.17-1.21)	1.13(1.11-1.15)	**<0.001**
ApoB, mg/L	0.76(0.74-0.79)	0.80(0.77-0.83)	0.82(0.79-0.85)	0.79(0.76-0.82)	0.094
LpA, mg/L	0.36(0.32-0.40)	0.46(0.42-0.49)	0.38(0.35-0.42)	0.40(0.37-0.44)	**0.001**
LDL cholesterol/ApoB ratio	3.87(3.64-4.10)	3.85(3.63-4.08)	4.07(3.85-4.30)	3.88(3.65-4.13)	0.481
tCys (μmol/L)	<219.5	219.5-250.2	250.3-284.1	>284.1	
Total cholesterol, mmol/L	4.73(4.64-4.82)	4.78(4.69-4.87)	4.76(4.67-4.85)	4.71(4.62-4.80)	0.675
Triglycerides, mmol/L	1.84(1.71-1.96)	1.93(1.81-2.05)	1.87(1.75-1.99)	1.83(1.70-1.94)	0.656
LDL cholesterol, mmol/L	2.98(2.91-3.07)	3.03(2.94-3.11)	3.02(2.94-3.10)	2.96(2.88-3.04)	0.685
HDL cholesterol, mmol/L	1.13(1.10-1.15)	1.08(1.05-1.11)	1.12(1.10-1.14)	1.10(1.07-1.13)	0.053
ApoA-I, mg/L	1.13(1.09-1.17)	1.14(1.10-1.18)	1.19(1.15-1.24)	1.12(1.08-1.17)	0.141
ApoB, mg/L	0.78(0.75-0.82)	0.80(0.77-0.83)	0.81(0.78-0.84)	0.79(0.76-0.82)	0.739
LpA, mg/L	0.44(0.40-0.47)	0.38(0.34-0.42)	0.41(0.38-0.45)	0.38(0.35-0.42)	0.073
LDL cholesterol/ApoB ratio	4.04(3.82-4.27)	3.80(3.58-4.03)	3.95(3.72-4.17)	3.89(3.67-4.12)	0.517

* Data are presented as mean (95% confidence interval). Analyses of covariance (ANCOVA) were performed using the general linear model approach after adjustment for age, gender, smoke, hypertension, fasting plasma glucose, and creatinine.

*P *values are significant as shown in bold.

Q1 = quartile 1, Q2 = quartile 2, Q3 = quartile 3, Q4 = quartile 4

**Figure 1:**
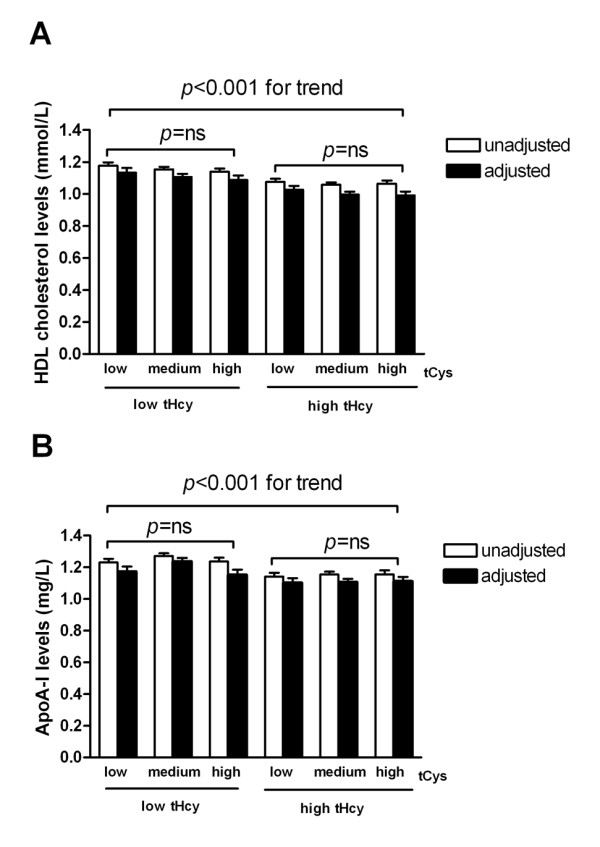
**Plasma HDL cholesterol and ApoA-I levels according to the different combinations of tHcy and tCys levels**. A. plasma HDL cholesterol levels. B. plasma ApoA-I levels. Analyses of covariance (ANCOVA) were performed using the general linear model within different combinations of tHcy and tCys. Bars are mean ± SEM, white bars are unadjusted, black bars are adjusted for age, gender, smoke, hypertension, fasting plasma glucose, and creatinine. Both *P *< 0.001 for trend, ns indicates no significant.

In an attempt to investigate a combination variable of tHcy and tCys in relation to plasma HDL cholesterol and ApoA-I levels (Figure 1), we found the lowest HDL cholesterol and ApoA-I concentrations in subjects with high tHcy and tCys. There were significant linear trend decrease of HDL cholesterol and ApoA-I concentrations in all of the 6 tHcy-tCys combination groups with and without adjusted for age, gender and other confounders. However, no significance was showed the changes of HDL cholesterol and ApoA-I levels between the subgroups of low, medium, and high tCys levels in both low and high tHcy levels.

### The association of plasma tHcy, tCys and the risk of CAD

**Figure 2:**
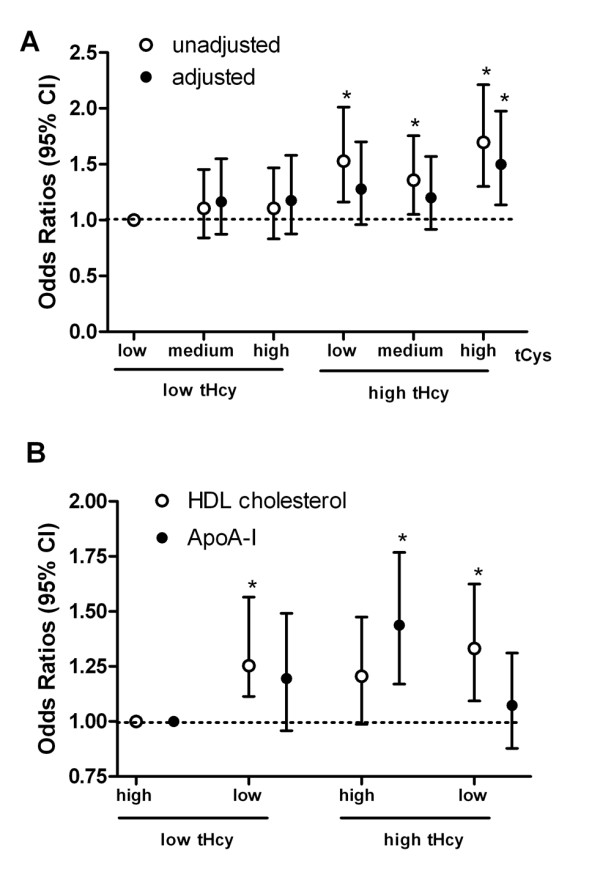
**Combined effects of plasma tHcy and tCys, HDL cholesterol or ApoA-I levels on the risk of coronary artery disease prevalence**. A. Within different combinations of tHcy and tCys, low levels of tHcy and tCys as reference group, odds ratios and 95% confidence interval are from logistic regression analysis with and without controlling for age, gender, smoke, hypertension, triglycerides, HDL, ApoA-I, fasting plasma glucose, creatinine. B. Subjects with combinations of low levels of tHcy and high levels of HDL cholesterol or ApoA-I served as the reference group. ORs and 95% CI are from logistic regression analysis with controlling for age, gender, smoke, hypertension, triglycerides, fasting plasma glucose, creatinine. * *P *< 0.05.

After additional adjustment for the age, gender and other potential confounders, plasma tCys showed no apparent association, high concentration of tHcy seemed to be associated with increased risk for CAD (OR: 1.34; 95% CI (1.04-1.74)) (Table 4). We investigated a combination variable of tHcy and tCys in relation to the CAD risk (Figure 2A). Notably, the combinations of high tHcy and low (OR: 1.528, 95% CI (1.161-2.011)), medium (OR: 1.358, 95% CI (1.05-1.755)), or high tCys (OR: 1.696, 95% CI (1.301-2.211)) all had significantly increased risk for CAD. However, after adjusted for age, gender and other confounders, only the combination of high tHcy and high tCys had a significantly increased risk for CAD (OR: 1.499, 95% CI (1.137-1.976)).

**Table 4:** Unadjusted and adjusted risk for CAD prevalence with increasing quartiles of tHcy and tCys levels

	Q1	Q2	Q3	Q4	*P *for trend
tHcy (μmol/L)	<9.1	9.1-12.3	12.4-16.5	>16.5	
CAD	228(44.5%)	255(49.9%)	280(53.8%)	290(56.3%)	
Unadjusted OR (95% CI)	1.0(referent)	1.24(0.97-1.58)	1.45(1.14-1.85)	1.61(1.26-2.05)	*P *< 0.001
Adjusted OR* (95% CI)	1.0(referent)	1.08(0.83-1.39)	1.12(0.87-1.44)	1.35(1.04-1.74)	*P *< 0.001
Adjusted OR^† ^(95% CI)	1.0(referent)	1.04(0.80-1.36)	1.14(0.88-1.47)	1.34(1.03-1.74)	*P *< 0.001
tCys (μmol/L)	<219.5	219.5-250.2	250.3-284.1	>284.1	
CAD	259(50.6%)	261(50.5%)	260(50.4%)	273(53.2%)	
Unadjusted OR (95% CI)	1.0(referent)	0.99(0.78-1.27)	0.98(0.77-1.26)	1.11(0.87-1.42)	NS
Adjusted OR* (95% CI)	1.0(referent)	1.04(0.81-1.34)	1.05(0.82-1.35)	1.16(0.90-1.49)	NS
Adjusted OR^† ^(95% CI)	1.0(referent)	1.02(0.79-1.32)	1.07(0.83-1.38)	1.17(0.91-1.52)	NS

* Adjusted for age and gender.

† Adjusted for age, gender, smoke, hypertension, triglycerides, HDL, ApoA-I, fasting plasma glucose, creatinine.

CI = confidence interval, OR = odds ratio, Q1 = quartile 1, Q2 = quartile 2, Q3 = quartile 3, Q4 = quartile 4.

### Combined effect of plasma tHcy and HDL, ApoA-I on the risk of CAD

In the correlation analysis, we found plasma tHcy were associated negatively with plasma HDL cholesterol and ApoA-I levels, further, we investigated a combination variable of tHcy and HDL cholesterol or ApoA-I in relation to the CAD risk (Figure 2B). Interestingly, low HDL cholesterol combined with low tHcy or high tHcy all had increased risk for CAD (OR: 1.254, 95% CI (1.114-1.565); OR: 1.332, 95% CI (1.093-1.624); respectively) whereas combination of high HDL cholesterol and high tHcy had no significant association with the risk of CAD. In respect of combinations of tHcy and ApoA-I, only the combination of high tHcy and high ApoA-I had an increased risk for CAD (OR: 1.438, 95% CI (1.170-1.768)).

## Discussion

In this cross-sectional study, our results showed significant negative relationship between plasma tHcy and HDL cholesterol or ApoA-I levels, positive dose-response relationship between plasma tHcy and the risk of CAD was observed. Furthermore, we found the joint effects of plasma tHcy and tCys decreased the plasma HDL cholesterol and ApoA-I levels, and increased the risk of CAD. In respect of joint effects of plasma tHcy and plasma HDL cholesterol or ApoA-I, low HDL cholesterol combined with both low and high tHcy increased the risk of CAD whereas only combination of high ApoA-I and high tHcy increased the risk of CAD.

Some reports showed a reduced expression of ApoA-I and decreased HDL cholesterol levels were observed in mice with genetically induced hyperhomocysteinemia. In the *methfr*^+/- ^deficient mice model, homocysteine reduced the expression of peroxisome proliferator-activated receptor (PPARα) and decreased the ApoA-I promoter activity and its protein levels [12]. In addition to its influence on ApoA-I, hyperhomocysteinemia inhibited reverse cholesterol transport by reducing circulating HDL via inhibiting apoA-I protein synthesis and enhancing HDL cholesterol clearance in the *cbs*^-/-^*apoe*^-/- ^mice [11]. Moreover, homocysteine associated with increased small size HDL3c suggest mechanisms related with the impaired synthesis of ApoA-I and HDL and abnormal maturations of HDL particles [18].

Previous studies showed plasma tCys is positively related to cholesterol, diastolic blood pressure and BMI in Hordaland homocysteine study [19,20], and positively correlated with fasting LDL cholesterol and ApoB in the COMAC cohort [8]. But in our present study, there was no significant association between plasma tCys and lipids levels. Because the blood samples in patients were drawn after the disease episode, however, we cannot rule out the possibility that tCys levels might be influenced by the disease itself. There is also the possibility that medication or change in lifestyle and dietary habits might have influenced the levels of tCys in our population [20]. In addition, several observations suggested that the role of cysteine in hepatic synthesis of ApoB may explain the epidemiology link of tCys and obesity [20-22].

Homocysteine and cysteine have been considered as risk factors of cardiovascular disease [8,23]. The studies concerning homocysteine lowering vitamin therapy did not mention the impact on cysteine levels [24,25]. We excluded the subjects who used vitamin supplement which might influence the plasma tHcy levels, but we can not rule out the effect of the dietary changes of the patients on the plasma tCys levels. It has been reported that large variations of cysteine levels in plasma which been observed in healthy subjects and this may be partly related to food intake[26]. Underlying nutrient disturbances may be an important determinant of plasma tCys levels. In addition, we did not select the health subjects as controls in our study population, this may be a selective bias of no association between plasma tCys and CAD.

Further, the relation between tHcy and CAD was evaluated for different combinations of tHcy and tCys concentrations. The subjects with high tHcys and high tCys levels had the highest ORs for CAD with and without adjustment for potential confounders. However, there was no relation for CAD combined low tHcy levels with medium and high tCys levels, this indicates that the effects of high tCys on the risk of CAD are dependent on the associated levels of tHcy[8]. Moreover, the subjects with high tHcys and high tCys levels also had the lowest HDL cholesterol and ApoA-I levels. This may suggest the synergistic effects of tHcy and tCys decreased the plasma HDL cholesterol and ApoA-I levels and increased the risk of CAD [7,27].

Next, we analyzed the joint effects of tHcy and HDL cholesterol or ApoA-I levels on the risk of CAD. Low HDL cholesterol levels combined with both low and high tHcy levels had significant ORs for CAD, it indicates that low HDL cholesterol levels was independent of plasma tHcy associated with the increased risk of CAD. On the contrary, the subjects with high HDL cholesterol levels and high tHcy levels had no significant OR for CAD, this may be explained that the protective effect of high HDL cholesterol levels could offset the harmful effect of high tHcy levels on the risk of CAD. On the other hand, the subjects with high ApoA-I levels and high tHcy levels did not have protective effect but increased the risk of CAD. Increased plasma tHcy levels correlated negatively with decreased ApoA-I and HDL cholesterol levels, and also positively with increased small size HDL3c [18]. So the subjects with high tHcy levels and high ApoA-I levels might have increased plasma small size HDL3c levels. This suggested the subjects with high tHcy levels which might impair the function of Apo-I and HDL and abnormal maturation of HDL particles although in presence of high ApoA-I levels would increase the risk of CAD.

## Conclusion

our cross-sectional data showed that the plasma tHcy rather than tCys was associated with decreased plasma HDL cholesterol and ApoA-I levels and the increased risk of CAD. Further, we found plasma tCys was dependent on and increased the synergistic effect of plasma tHcy. However, plasma HDL cholesterol was independently and counteracted the effect of plasma tHcy. High plasma tHcy was associated with increased risk of CAD although in presence of high ApoA-I levels. Studies on the joint effects of plasma tHcy and other risk factors on the risk of CAD are therefore underway.

## Abbreviations

tHcy: total homocysteine; tCys: total cysteine; CAD: coronary artery disease; BMI: body mass index; ApoA-I: apolipoprotein A-I; ApoB: apolipoprotein B; HDL: high density lipoprotein; LDL: low density lipoprotein; LpA: lipoprotein A.

## Competing interests

The authors declare that they have no competing interests.

## Authors' contributions

Conduct of the study: YZ, XL, DS,

Design and manuscript writing: YX, WL, JM

Data collection and analysis: DL, MX, JQ

All authors have read and approved the final manuscript.

## Funding Sources

This work was supported by grant from the Key Project (No.30730079) and grant (No.30872101) of National Natural Science Foundation of China.
